# Parallel administration of nanoliposomal irinotecan and *levo*-leucovorin for pancreatic cancer

**DOI:** 10.1186/s12885-023-11205-6

**Published:** 2023-07-31

**Authors:** Ryoji Takada, Kenji Ikezawa, Takuo Yamai, Ko Watsuji, Yusuke Seiki, Yasuharu Kawamoto, Takeru Hirao, Sena Higashi, Makiko Urabe, Yugo Kai, Tasuku Nakabori, Hiroyuki Uehara, Michiyo Kotani, Toshinari Yagi, Miho Kimura, Keisuke Nozaki, Mari Takagi, Kazuyoshi Ohkawa

**Affiliations:** 1 Department of Hepatobiliary and Pancreatic Oncology, Osaka International Cancer Institute, 3-1-69 Otemae, Chuo-Ku, Osaka, 541-8567 Japan; 2 Department of Nursing, Osaka International Cancer Institute, Osaka, Japan; 3 Department of Outpatient Chemotherapy, Osaka International Cancer Institute, Osaka, Japan; 4 Department of Pharmacy, Osaka International Cancer Institute, Osaka, Japan

**Keywords:** Pancreatic cancer, Nanoliposomal irinotecan, *Levo*-leucovorin, Chemotherapy

## Abstract

**Background:**

Nanoliposomal irinotecan (nal-IRI) plus 5-fluorouracil (5-FU)/*levo*-leucovorin (*Levo*-LV) was approved for unresectable pancreatic cancer (UR-PC) in March 2020 in Japan. *Levo*-LV is administered by intravenous infusion over 120 min following 90 min intravenous infusion of nal-IRI (conventional method), causing a significant burden on both patients and the outpatient chemotherapy room owing to the prolonged administration time. Thus, from July 2021, we introduced the simultaneous intravenous administration of nal-IRI and *Levo*-LV (parallel method) with the approval of the institutional regimen committee.

**Methods:**

We retrospectively reviewed the data of 69 patients with UR-PC who received nal-IRI plus 5-FU/*Levo*-LV at our hospital between June 2020 and October 2021. We examined the safety of the parallel method and compared the treatment outcomes and administration times between the two methods.

**Results:**

The median age was 66 years (54%, male). Disease statuses were locally advanced, metastatic, and postoperative recurrence after pancreatectomy in 7, 50, and 12 patients, respectively. Nal-IRI plus 5-FU/*Levo*-LV treatment was second and third-line or later in 35 and 34 patients, respectively. No intravenous line problems were observed during the parallel administration of nal-IRI and *Levo*-LV. Although there were no significant differences in response rates and adverse events between the two methods, the administration time was significantly shorter in the parallel method than in the conventional method.

**Conclusion:**

The parallel administration of nal-IRI and *Levo*-LV is clinically safe and not inferior in efficacy. Moreover, parallel administration may offer convenience to patients and healthcare workers by reducing administration time.

## Background

In the past decade, several chemotherapy regimens have been developed worldwide, resulting in improved outcomes for various malignancies [1–3]. A prevailing trend involves the use of multidrug combination therapies, including cytotoxic chemotherapeutic agents, molecular targeted therapy, and immunotherapy, which often exhibit superior efficacy to single-agent therapies [4]. In the chemotherapy for pancreatic cancer (PC), combination therapies, including leucovorin (LV) calcium (folinic acid), fluorouracil, irinotecan hydrochloride, and oxaliplatin (FOLFIRINOX) and gemcitabine plus nab-paclitaxel, have become standard treatment options for unresectable (UR) PC (metastatic or locally advanced) [5–11].

The NAPOLI-1 trial, a global randomized phase III trial, demonstrated that nanoliposomal irinotecan (nal-IRI) in combination with 5-fluorouracil (5-FU)/LV improved the survival of patients with metastatic PC refractory to gemcitabine-based chemotherapy [12]. As the use of LV to enhance the effects of 5-FU has not been approved in Japan, *Levo*-LV was used in a domestic phase II trial, and the combination of nal-IRI plus 5-FU/*Levo*-LV was subsequently approved in Japan in March 2020 [13].

Folinic acid contains *dext*-rotatory and *levo*-rotatory isomers, with only the latter being pharmacologically active [14]. *Levo*-LV, folinic acid of *levo*-rotatory isomer, is the pure active form of calcium LV. *Levo*-LV is generally administered by intravenous infusion over 120 min, whereas LV is administered over 30–120 min [15]. The nal-IRI plus 5-FU/*Levo*-LV regimen consists of 70 mg/m^2^ nal-IRI administered by intravenous infusion over 90 min, followed by 200 mg/m^2^ *Levo*-LV by intravenous infusion over 120 min, and finally, 2400 mg/m^2^ 5-FU by continuous intravenous infusion over 46 h, every 2 weeks [13, 16]. Therefore, patients who receive the nal-IRI plus 5-FU/*Levo*-LV regimen must spend more than 210 min in the outpatient chemotherapy room on day 1 of the regimen. In addition, it is operationally inefficient because chemotherapeutic regimens with long administration times occupy beds in the outpatient chemotherapy room, requiring healthcare workers to spend a long time in the hospital.

Although *Levo*-LV and irinotecan are administered in parallel in regimens such as FOLFIRINOX and FOLFIRI, there have been no reports on the simultaneous administration of nal-IRI and *Levo*-LV [6, 17]. Therefore, this study aimed to investigate the clinical safety of the parallel administration of nal-IRI and *Levo*-LV and to compare the treatment outcomes and administration times between conventional and parallel administrations.

## Methods

This study retrospectively reviewed consecutive patients with UR-PC, who received nal-IRI plus 5-FU/*Levo*-LV chemotherapy at Osaka International Cancer Institute between June 2020 and January 2022. The major inclusion criteria were as follows: (1) histologically or cytologically proven pancreatic ductal adenocarcinoma; (2) unresectable status including metastatic PC, locally advanced PC, and recurrence after pancreatectomy according to Union for International Cancer Control TNM classification 8th edition; (3) no prior chemotherapy regimen of nal-IRI plus 5-FU/Levo-LV; and (4) adequate functioning of major organs. Patients with other active cancers or those who received nal-IRI plus 5-FU/*Levo*-LV regimen with both conventional and parallel administration methods during the observation period were excluded from this study.

For each patient, we collected data regarding age, sex, Eastern Cooperative Oncology Group (ECOG) performance status (PS), tumor status, treatment line, biliary drainage, and uridine diphosphate glucuronosyltransferase 1A1 (UGT1A1) status. This study was approved by the Institutional Regimen Committee and Institutional Review Board of Osaka International Cancer Institute (22,004–4) and was conducted in accordance with the Declaration of Helsinki. The requirement for informed consent was waived owing to the retrospective nature of this study.

### Treatment

The conventional administration method of nal-IRI plus 5-FU/*Levo*-LV regimen (conventional method) consisted of 80 mg/m^2^ nal-IRI (irinotecan hydrochloride trihydrate salt, equivalent to 70 mg/m^2^ irinotecan free base) administered by intravenous infusion over 90 min, followed by intravenous infusion of 200 mg/m^2^ *Levo*-LV over 2 h, and continuous infusion of 2400 mg/m^2^ 5-FU over 46 h every 2 weeks, according to a previously reported protocol [13]. The simultaneous administration of nal-IRI plus 5-FU/*Levo*-LV regimen consisted of parallel intravenous administration of 200 mg/m^2^ *Levo*-LV over 2 h and 80 mg/m^2^ nal-IRI over 90 min, followed by continuous infusion of 2400 mg/m^2^ 5-FU over 46 h every 2 weeks. In this study, this administration method was referred to as the parallel method. Prior to chemotherapy, palonosetron hydrochloride, a 5-hydroxytryptamine receptor antagonist, and dexamethasone were generally administered as prophylactic antiemetics. If patients experienced severe nausea, a neurokinin-1 receptor antagonist (aprepitant) was additionally administered according to the physician’s discretion. The dosage of each drug was adjusted by the physicians based on adverse events (AEs), patient comorbidities, and patient conditions, including age, PS, tumor status, treatment line, and UGT1A1 status. The treatment was continued until disease progression, unacceptable toxicity, patient refusal, or discontinuation as decided by the physicians.

### Evaluation of treatment outcomes and administration time

We evaluated AEs occurring within 90 days of initiation of the nal-IRI plus 5-FU/*Levo*-LV regimen or until treatment discontinuation if it occurred within 90 days. Hematological and non-hematological AEs were classified according to the Common Terminology Criteria for Adverse Events version 5.0. Tumor response was assessed in accordance with the revised Response Evaluation Criteria in Solid Tumors guidelines (version 1.1), with the best response from the initiation of nal-IRI plus the 5-FU/*Levo*-LV regimen. The relative dose intensity (RDI) was evaluated during the first administration. The percentage dose of each drug was calculated by dividing the actual dose with the full dose. Administration time was defined as the time from initiation of the first medication to connection with the 5-FU infusion pump during the first use of the outpatient chemotherapy room on day 1 of the nal-IRI plus 5-FU/*Levo*-LV regimen.

### Statistical analyses

We used the Mann–Whitney U test to compare baseline characteristics for continuous variables and the chi-squared or Fisher’s exact tests for categorical variables between groups. We compared the RDI and administration time between groups using a t-test for continuous variables. Statistical analyses were performed using EZR (Saitama Medical Center, Jichi Medical University, Saitama, Japan), a graphical interface for the R Commander software package for Windows (version 1.53) [18]. *P*-value < 0.05 was considered statistically significant.

## Results

### Patient characteristics

Table 1 summarizes the characteristics of 69 patients included in the present study with a median age of 66 (range, 37–81) years; among them, 37 (53.6%) patients were male. The ECOG PS was zero in 33 (47.8%), one in 35 (50.7%), and two in one (1.5%) patients. The tumor statuses were locally advanced in seven (10.1%), metastatic in 50 (72.5%), and postoperative recurrence after pancreatectomy in 12 (17.4%) patients. The treatment lines were second and third-line or later in 35 (50.7%) and 34 (49.3%) patients, respectively. Regarding biliary obstruction caused by PC, 44 (63.7%) patients underwent endoscopic biliary drainage and six (8.7%) patients underwent biliary anastomosis after pancreatoduodenectomy. The UGT1A1 statuses were as follows: wild-type for UGT1A1*6 and UGT1A1*28 in 25 (36.2%) patients, heterozygous for UGT1A1*6 or UGT1A1*28 in 35 (50.7%) patients, double-variant heterozygous for UGT1A1*6 or UGT1A1*28 in four (5.8%) patients, and homozygous for UGT1A1*6 or UGT1A1*28 in five (7.3%) patients. The major organ functions at the initiation of nal-IRI plus 5-FU/*Levo*-LV are summarized in Table 2.

**Table 1:** Baseline characteristics of the study patients and comparison between the conventional and parallel methods

	All	Conventional method	Parallel method	*P*-value
	(*n* = 69)	(*n* = 49)	(*n* = 20)	
Age, years (median, range)	66 (37–81)	66 (45–81)	64 (37–79)	0.989
Sex				
Male/female	37/32	26/23	11/9	1
ECOG PS				
0/1/2	33/35/1	25/24/0	8/11/1	0.251
Tumor status				
Locally advanced/metastatic/recurrence	7/50/12	4/38/7	3/12/5	0.338
Treatment line				
2nd/ ≥ 3rd	35/34	25/24	10/10	1
Biliary drainage				
Yes/no/anastomosis	44/19/6	32/13/4	12/6/2	0.922
UGT1A1 (*6/*28)				
Wild/wild	25	21	4	0.712
Wild/heterozygous	16	8	8	
Heterozygous/wild	19	14	5	
Heterozygous/Heterozygous	4	1	3	
Homozygous/wild	3	3	0	
Wild/homozygous	2	2	0	

*ECOG PS* Eastern Cooperative Oncology Group performance status, *UGT1A1* Uridine diphosphate glucuronosyltransferase 1A1

Statistical significance at *P* < 0.05

**Table 2:** Baseline major organ functions of the study patients and comparison between the conventional and parallel methods

	All	Conventional method	Parallel method	*P*-value
	(*n* = 69)	(*n* = 49)	(*n* = 20)	
WBC (median, range)	5450 (1930–13190)	5080 (1930–12230)	5795 (1990–13190)	0.543
NEU	3160 (1010–11080)	3040 (1010–10260)	3795 (1040–11080)	0.394
Hb	10.7 (8.1–13.8)	10.8 (8.7–13.8)	10.6 (8.1–12.7)	0.389
PLT	23.6 (6.6–58.3)	25.0 (6.6–58.3)	18.3 (9.7–45.1)	0.916
Alb	3.5 (2.3–4.5)	3.5 (2.7–4.5)	3.6 (2.3–4.2)	0.868
T-bil	0.5 (0.2–2.8)	0.5 (0.3–2.0)	0.5 (0.2–2.8)	0.698
AST	21 (12–77)	22 (12–77)	21 (12–54)	0.869
ALT	18 (4–78)	17 (4–78)	18 (8–52)	0.853
Cre	0.66 (0.45–1.37)	0.67 (0.48–1.37)	0.61 (0.45–1.10)	0.144

*WBC* White blood cell, *NEU* Neutrophil, *Hb* Hemoglobin, *PLT* Platelet, *Alb* albumin, *T-bil* Total bilirubin, *AST* Aspartate aminotransferase, *ALT* Alanine aminotransferase, *Cre* Creatinine

Statistical significance at *P* < 0.05

Of the 69 patients who received nal-IRI plus 5-FU/*Levo*-LV, 49 (71.0%) received these medications using the conventional method and 20 (29.0%) received these medications using the parallel method. Table 1 shows a comparison of the patient characteristics between the conventional and parallel method groups. No significant differences were observed in age, sex, ECOG PS, tumor status, treatment line, biliary drainage, and UGT1A1 status. There were no significant differences in white blood cell count, neutrophil count, hemoglobin level, platelet count, albumin level, total bilirubin level, aspartate aminotransferase level, alanine aminotransferase level, and creatinine level between the two groups.

### Treatment outcomes and administration time

In terms of RDI, there was no significant difference between the conventional and parallel methods (5-FU: mean ± standard deviation [SD], 86.1% ± 17.7 and 84.9% ± 17.2, *P* = 0.790; *Levo*-LV: 98.1% ± 8.1 and 100.0% ± 0, *P* = 0.299; nal-IRI: 86.1% ± 16.7 and 82.1% ± 16.9, *P* = 0.373, respectively). No intravenous line problems, including macroscopic cloudiness or occlusion by crystallization, were observed during the parallel administration of *Levo*-LV and nal-IRI. Table 3 presents a comparison of the AEs of the two methods. The most frequently observed AEs ≥ grade 3 were leukopenia and neutropenia in both methods. Although no treatment-related deaths were reported, one case of febrile neutropenia occurred with each method, one case of pneumonitis occurred with the conventional method, and two cases of pneumonitis occurred with the parallel method. No significant differences in AEs were observed between the two methods.

**Table 3:** A comparison of adverse events between the conventional and parallel methods

	Conventional method		Parallel method		*P*-value
	(*n* = 49)		(*n* = 20)		
	≥ grade 3		≥ grade 3		
	n	%	n	%	
Hematological toxicities					
Leukopenia	9	18.4	7	35.0	0.207
Neutropenia	13	26.5	7	35.0	0.562
Thrombocytopenia	0	0	0	0	1.000
Anemia	7	14.3	5	25.0	0.309
Febrile neutropenia	1	2.0	1	5.0	0.499
Non-hematological toxicities					
Anorexia	9	18.4	2	10.0	0.490
Fatigue	3	6.1	2	10.0	0.623
Nausea	3	6.1	0	0	0.551
Vomiting	1	2.0	0	0	1.000
Diarrhea	4	8.2	0	0	0.315
Elevated T-bil	0	0	0	0	1.000
Elevated ALT	4	8.2	2		1.000
Elevated AST	0	0	2	10.0	0.081
Elevated creatinine	0	0	1	5.0	0.290
Pneumonitis	1	2.0	2	10.0	0.199

*T-bil* Total bilirubin, *AST* Aspartate aminotransferase, *ALT* Alanine aminotransferase

Statistical significance at *P* < 0.05

Regarding treatment efficacy, we evaluated response and disease control rates. There was no significant difference between the conventional and parallel methods (response rate: 8.2% vs. 7.2%, *P* = 1.00; disease control rate: 42.9% vs. 46.4%, P = 0.43, respectively) (Table 4).

**Table 4:** Comparison of response and disease control rates between the conventional and parallel methods

	All		Conventional method		Parallel method		*P*-value
	(*n* = 69)		(*n* = 49)		(*n* = 20)		
Best overall response	n	%	n	%	n	%	
Complete response	0		0		0		
Partial response	5		4		1		
Stable disease	27		17		10		
Progressive disease	26		20		6		
Not evaluated	11		8		3		
Response rate	5	7.2	4	8.2	1	7.2	1.00
Disease control rate	32	46.4	21	42.9	11	46.4	0.43

Statistical significance at *P* < 0.05

**Fig. 1:**
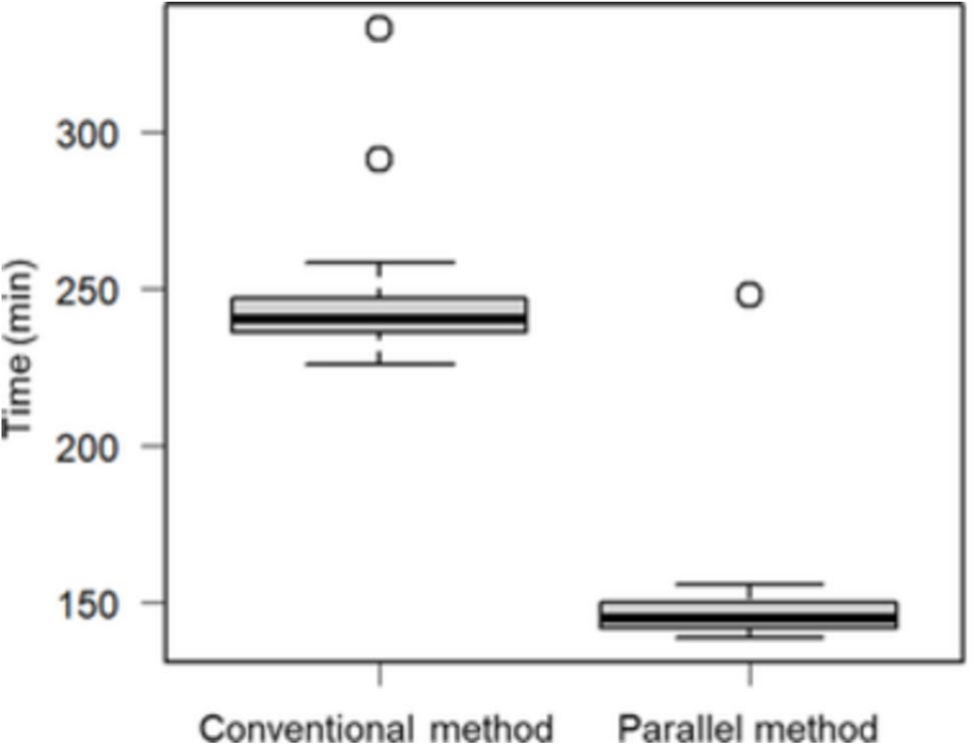
Comparison of administration time between the conventional and parallel methods

Finally, we compared the administration time between the two groups. The mean administration time was significantly shorter in the parallel method (151 min, SD = 24.7 min) than in the conventional method (245 min, SD = 18.6 min) (*P* < 0.001) (Fig. 1).

## Discussion

Second-line chemotherapy has been shown to improve the prognosis of patients with UR PC [12, 19–23]. In a randomized global phase III trial, nal-IRI plus 5-FU/LV demonstrated superiority over 5-FU/LV as second-line chemotherapy for patients with metastatic PC. However, in Japan, *Levo*-LV was used as a replacement for LV because it was not approved to enhance the effects of 5-FU [12]. LV can be administered for 30 min, whereas *Levo*-LV must be administered for 120 min [15]. In a phase II study of nal-IRI plus 5-FU/*Levo*-LV in Japan, nal-IRI and *Levo*-LV were sequentially administered, which is the conventional method used in this study [13]. However, this method poses a burden on both patients and the outpatient chemotherapy room because it takes a long time to administer *Levo*-LV (120 min) following nal-IRI (90 min). Therefore, we conducted a parallel study with the approval of the Institutional Regimen Committee of our hospital. This retrospective study is the first report on the safety of the parallel administration of nal-IRI and *Levo*-LV (parallel method in this study).

In all patients, we were able to safely administer nal-IRI and *Levo*-LV during parallel infusion without catheter occlusion or crystallization of the infusion lines. The high solubility of *Levo*-LV enables its safe mixing with various drugs [23]. The incidence of major AEs (≥ grade 3) was not significantly different between the two methods (Table 3). Similarly, there was no significant difference in the response rates between the two methods, indicating short-term efficacy (Table 4). However, as shown in Fig. 1, the administration time was significantly shorter in the parallel method than in the conventional method (*P* < 0.001). These results suggest that the parallel method significantly decreases the administration time (approximately 90 min) without increasing the incidence of AEs or intravenous line problems.

For patients with a poor prognosis, spending a significant amount of time on chemotherapy can limit daily life and reduce quality of life. Moreover, the shortening of chemotherapy time can help reduce the load on beds/chairs in outpatient chemotherapy room and healthcare workers, including medical doctors, nurses, pharmacists, and office workers [24, 25]. Efficient use of limited resources is necessary because the human and physical resources available to hospitals cannot be easily increased. Therefore, we believe that our approach is important for both patients and healthcare workers.

This study has some limitations. First, although this was a nonrandomized and retrospective study comparing different administration methods of the same treatment performed in different periods at a single center, there was no significant difference in patient characteristics between the two methods. Second, we did not perform microscopic or pharmacokinetic examinations in parallel administration of nal-IRI and *Levo*-LV. Although there have been no reports on this, we did not observe infusion line problems [17].

In conclusion, the parallel administration of nal-IRI and *Levo*-LV is clinically safe and not inferior in efficacy in patients with UR-PC. Our results suggest that the parallel administration of nal-IRI and *Levo*-LV may offer convenience to patients and healthcare workers by reducing administration time.

## Data Availability

The data presented in this study are available on request from the corresponding author. The data are not publicly available due to privacy issues.
